# Supplementary material for: Content-rich biological network constructed by mining PubMed abstracts
Source: BMC Bioinformatics. 2004 Oct 8;5:147. doi: 10.1186/1471-2105-5-147 (PMC528731; doi:10.1186/1471-2105-5-147)
Supplement: Additional File 2 — The original results of the above study (non-essential files are deleted to keep the file size under the limit set by BMC bioinformatics). [file 1471-2105-5-147-S2.bz2 › chilibotAdditionalFile2/dip05/683ID10906130E17998/html/RPOA_RPOB.html]

 


 **RPOA** and **RPOB** 
  
Found 22 abstracts in PubMed, retrieved 05.  
 

 What does Google say? 
 PDF only 
| .edu only 

---

**Interactive relationship** (e.g. stimulation, inhibition, etc)

**Inhibitory relationship**- The lack of a PEP  [ **RPOA** ]  promoter in these species coincides with the loss of two genes that encode subunits of PEP  [ **RPOA** ]   **rpoA**  and  **rpoB** .  Ref: 12624769 Planta, 2003
**Neutral relationship**

**Non-interactive relationship** (e.g. studied together, co-existance, homology, etc.)

- Plastid run on assays and Northern analyses were employed to analyse gene expression in tobacco mutant plastids lacking the PEP  [ **RPOA** ]  genes  **rpoA** ,  **rpoB**  or rpoC1.  Ref: 10954088 Mol Gen Genet, 2000
- Plastid transcription in the holoparasitic plant genus Cuscuta parallel loss of the rrn16 PEP  [ **RPOA** ]  promoter and of the  **rpoA**  and  **rpoB**  genes coding for the plastid encoded RNA polymerase.  Ref: 12624769 Planta, 2003
- and group 3, SigF to SigI and each RNAP core subunit  **RpoA** ,  **RpoB** , RpoC1 and RpoC2 were overproduced and purified from Escherichia coli cells, then polyclonal antibodies were prepared.  Ref: 12527296 J Mol Biol, 2003
- Northern analysis of  **rpoA** ,  **rpoB** , rpoC, and six ribosomal protein genes indicated induction of transcription as well as antitermination as part of the mechanism of the regulation of gene expression.  Ref: 11681206 Proteomics, 2001
